# Transport of Prions in the Peripheral Nervous System: Pathways, Cell Types, and Mechanisms

**DOI:** 10.3390/v14030630

**Published:** 2022-03-18

**Authors:** Sam M. Koshy, Anthony E. Kincaid, Jason C. Bartz

**Affiliations:** 1 Department of Medical Microbiology and Immunology, School of Medicine, Creighton University, Omaha, NE 68178, USA; samkoshy@creighton.edu; 2 Department of Pharmacy Science, School of Pharmacy and Health Professions, Creighton University, Omaha, NE 68178, USA; anthonykincaid@creighton.edu

**Keywords:** prion, pathogenesis, transport, nerves

## Abstract

Prion diseases are transmissible protein misfolding disorders that occur in animals and humans where the endogenous prion protein, PrP^C^, undergoes a conformational change into self-templating aggregates termed PrP^Sc^. Formation of PrP^Sc^ in the central nervous system (CNS) leads to gliosis, spongiosis, and cellular dysfunction that ultimately results in the death of the host. The spread of prions from peripheral inoculation sites to CNS structures occurs through neuroanatomical networks. While it has been established that endogenous PrP^C^ is necessary for prion formation, and that the rate of prion spread is consistent with slow axonal transport, the mechanistic details of PrP^Sc^ transport remain elusive. Current research endeavors are primarily focused on the cellular mechanisms of prion transport associated with axons. This includes elucidating specific cell types involved, subcellular machinery, and potential cofactors present during this process.

## 1. Introduction

Prion diseases are protein misfolding disorders that can have an infectious, sporadic, or genetic etiology, and lead to the slow, progressive, and inevitable demise of the host through neuronal degeneration of the CNS [1,2,3]. Although prion diseases were initially identified early in the 20th century, the exact nature of the infectious agent responsible for such disorders has only recently been determined. Initially described as slow viruses, prion diseases were thought to be caused by a slowly progressive viral infection [4,5,6,7]. It is now established that prion diseases are caused by a proteinaceous infectious particle, or prion [1,8,9]. Importantly, prions do not contain a nucleic acid genome like viral particles, but have templating abilities that allow propagation in the host [10,11].

The prion protein (PrP^C^) is an endogenous protein coded by the PRNP gene that is present within most mammalian species with paralogues in certain reptiles such as turtles [12]. Although the exact functions of PrP^C^ are unknown, there is evidence that it participates in a variety of roles including neurogenesis, neuronal development, and synaptic function [13,14,15,16]. In the infectious prion disease etiology, PrP^C^ undergoes an aberrant conformational change into misfolded prion protein (PrP^Sc^) that is induced by the interaction of exogenous PrP^Sc^ with endogenous PrP^C^. In sporadic prion diseases PrP^C^ is thought to spontaneously adopt the self-propagating infectious PrP^Sc^ conformation. In familial forms of prion diseases, mutations in the PRNP gene result in an increased propensity of PrP^C^ adopting the PrP^Sc^ conformation compared to wild type PrP^C^ [17,18]. The tertiary and/or quaternary structure of PrP^Sc^ results in an increased resistance to degradation by proteases, heat, pH, and various other environmental factors compared to PrP^C^ [19,20,21,22,23,24,25]. In addition, whereas PrP^C^ is considered a monomer, PrP^Sc^ can aggregate within cells leading to cellular dysfunction, neurodegeneration, gliosis, and spongiosis [2,3,17,26,27]. This CNS pathology is what ultimately leads to the clinical signs of disease (ataxia, tremor, behavioral changes, lethargy, etc.) and the death of the host.

The structural conformation of PrP^Sc^ is hypothesized to encode prion strain diversity [28]. Prion strains are operationally defined as heritable strain-specific phenotypes of disease that are characterized by incubation period, clinical signs of disease, CNS pathology, and distribution of prions in the host [29,30]. Prion strain-specific biochemical and biological properties of PrP^Sc^ can include electrophoretic mobility, stability of PrP^Sc^ in the presence of protein denaturants, PrP^Sc^ aggregate size distribution, and rate of PrP^Sc^ formation [30,31,32,33]. It is unclear how these differences in strain-specific properties of PrP^Sc^ result in differences in the phenotype of disease. Recent work, however, suggests that PrP^Sc^ particle size can influence prion formation efficiency and PrP^Sc^ clearance and that strain-specific ratios of these PrP^Sc^ particle sizes may provide a mechanistic basis for strain-specific rates of prion formation, incubation period of disease, and tissue tropism [34,35,36]. 

Prion diseases affect a wide variety of mammalian species. Notable examples of prion diseases with an infectious etiology include kuru, variant Creutzfeldt Jacob disease (vCJD), bovine spongiform encephalopathy (BSE), transmissible mink encephalopathy (TME), chronic wasting disease (CWD), and scrapie. Kuru occurs in the Fore people of Papa New Guinea and is thought to be caused by the consumption of nervous tissues from individuals afflicted with CJD via a practice of mortuary feasts of deceased family members [27,37,38,39,40,41,42,43,44,45]. BSE was first described in the United Kingdom in 1987 [46] and was propagated in cattle by the feeding of BSE-tainted meat and bone meal back to cattle [47,48]. The BSE epidemic caused a public health crisis in Europe, resulting in the transmission of BSE to humans through the emergence of vCJD [49,50,51]. TME is a prion disease of ranch-raised mink that was initially thought to be caused by feeding mink scrapie-infected sheep tissue [52,53,54]. Epidemiological and experimental evidence from the Stetsonville outbreak of TME, however, suggested that TME is caused by feeding mink downer cattle infected with an unrecognized BSE-like disease [55,56]. Subsequent studies supporting this hypothesis showed that atypical sporadic L-type BSE is the source of TME [57]. CWD is an emerging prion disease of cervids that was first described in a captive deer facility in Colorado in 1969 and was officially characterized as a prion disease in 1980 [58,59]. CWD has continued to spread throughout the United States and North America and is currently found in 27 states and 3 Canadian provinces [60,61]. Recently, CWD cases have been identified in Norway, Sweden, and Finland. Compared to North American CWD, the Scandinavian CWD cases have PrP^Sc^ and transmission properties suggesting that it is a unique strain of CWD [24,62,63,64,65,66,67]. Notably, CWD prions persist in the environment and are highly contagious resulting in horizontal transmission in both captive and free-ranging cervid populations [24,25,68]. Scrapie in sheep and goats was described as early as the 18th century and is still present in sheep and goat populations today [7,37,69,70,71,72,73,74,75,76]. Regarded as the prototypical prion disease, scrapie has played a large role in understanding prion disease pathogenesis. Like CWD, it can persist in the environment and spread through horizontal transmission [77,78,79]. Notably, placentas of scrapie positive ewes contain prion infectivity and may be a source of infection in utero (vertical transmission) or through environmental contamination early in the lambing stages [80]. 

## 2. Prions Spread along Defined Neuroanatomical Pathways

### 2.1. Intraocular Inoculation of Prions

**Figure 1:**
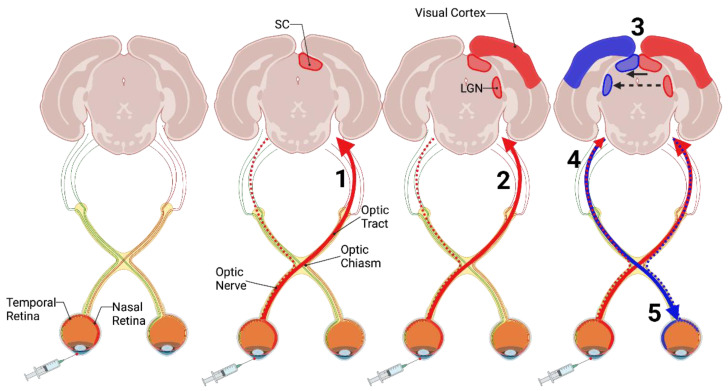
Prion spread following intraocular inoculation. Inoculation of prions into the retina of rodents results in anterograde spread of prion agent from the inoculated retina to the contralateral superior colliculus (SC; 1) and lateral geniculate nucleus (LGN), and visual cortex (solid red line and red structures; 2). As the animal approaches clinical endpoint, the ipsilateral SC, LGN, and visual cortex are also affected to a lesser extent (blue structures). This is thought to be due to reciprocal connections between the contralateral SC (solid black arrow; 3) and LGN (dashed black arrow; 3) and ipsilateral anterograde spread from the inoculated retina (dashed red line; 4). The uninoculated optic nerve and retina are also affected through retrograde prion spread (solid blue line; 5). Image created with BioRender.com, accessed on 28 February 2022.

Prions spread along defined neuroanatomical pathways following the intraocular route of infection [81,82]. Evidence that the prion agent is transported in CNS tissues via known neural pathways was established by determining the temporal and spatial spread of prion replication and pathology following intraocular injection of mice with the scrapie agent. Inoculation of the retina resulted in detection of prion infectivity in the contralateral superior colliculus (SC) via the optic nerve (Figure 1, [81,83]). In addition to the contralateral SC, scrapie infectivity was also identified in the contralateral lateral geniculate nucleus (LGN) and the contralateral visual cortex at later time points post-infection (Figure 1, [82,84]). Importantly, infectivity and vacuolation pathology was greater in the contralateral SC and LGN compared to the ipsilateral SC and LGN [82,85]. Since the rodent visual pathways almost completely decussate [86,87,88], this suggested the transport of prions from the injection site in the eye to the CNS occurs along defined neuroanatomical tracks. To support this hypothesis, enucleation of the retina just prior to or immediately after scrapie inoculation delayed the onset of clinical signs of disease, the increase in infectivity, and the development of vacuolation pathology [85]. This observation indicates that when the retina was removed, scrapie prions were not able to effectively access CNS structures as the neuronal pathway was eliminated. Following this centripetal (periphery to CNS) prion spread, a centrifugal (CNS to periphery) prion spread was also observed. 

At later timepoints post infection, the uninoculated contralateral optic nerve and retina developed scrapie infectivity [81] indicating prions spread from the peripheral structures to central structures, and then spread back into unaffected peripheral structures (Figure 1). Since this phenomenon is observed later in the disease process it may be less efficient compared to the initial spread of infectivity. As the major neuronal connections of the inoculated retina are to the contralateral SC and LGN [82,86], scrapie prions will more easily spread along these pathways and lead to earlier and more significant pathology. In contrast, the projection to the ipsilateral SC and LGN is more modest from the inoculated retina, and there may also be minor connections between the bilateral SC and LGN structures allowing for attenuated prion spread between the structures [86,89,90,91]. This results in prion invasion and the spread of pathology that takes longer (via more minor neuronal connections) and is less prominent at the end stage of disease. Once infected, the ipsilateral SC and LGN can transport prion infectivity to the uninoculated retina and its respective optic nerve via retrograde transport or the ipsilateral visual cortex via anterograde transport (Figure 1). Overall, these data indicate the anterograde transport of scrapie prions through established neuroanatomical pathways to central structures that can then spread contiguously or through other neuroanatomical connections within the CNS.

### 2.2. Extraneural Inoculation of Prions

**Figure 2:**
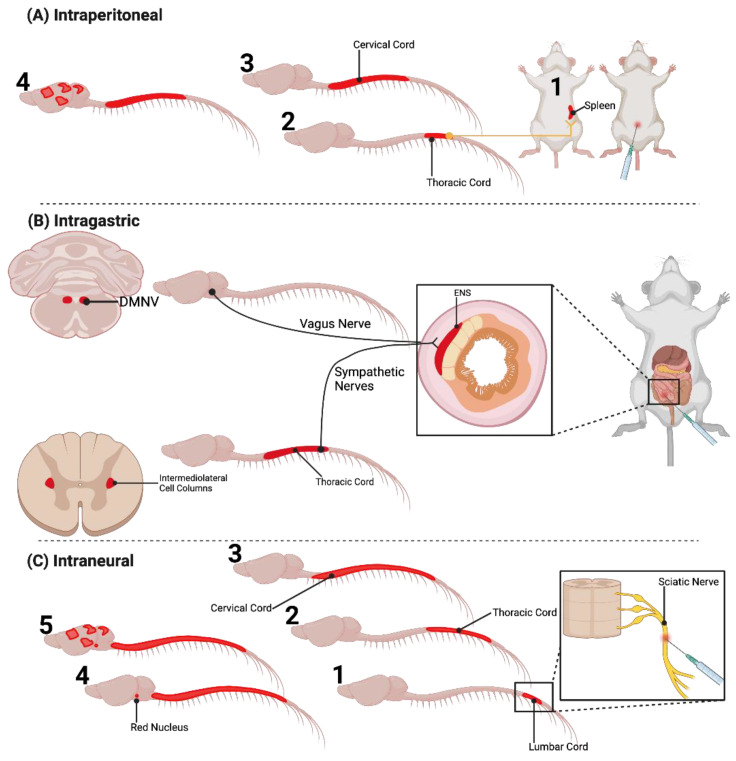
Prions spread along defined neuroanatomical pathways. (**A**) Prions inoculated through the intraperitoneal route (i.p.) initially accumulate in the spleen (1) before spreading along autonomic nerves into the thoracic spinal cord (2). Subsequent spread is rostrally within the spinal cord (3) to the brainstem and eventually into the brain (4). (**B**) Intragastric prion inoculation results in PrP^Sc^ accumulation in the myenteric plexus of the enteric nervous system (ENS) then subsequent spread along sympathetic efferent nerves to the thoracic spinal cord or directly to the dorsal motor nucleus of the vagus (DMNV) via parasympathetic vagal efferents. As with i.p. routes, spread occurs rostrally within the spinal cord to the brain. (**C**) Intraneural infection of prions results in spread along defined neuroanatomical pathways. In the case of the sciatic nerve, prion spread occurs along nerve roots to the VMNs of the lumbar spinal cord (1). Subsequent spread of prions in the spinal cord occurs in the rostral direction (2 and 3) toward brainstem nuclei that include the red nucleus (RN), vestibular nucleus, and cortex (4 and 5). Image created with BioRender.com, accessed on 28 February 2022.

Prions spread along defined neuroanatomical pathways following extraneural routes of infection. Intraperitoneal (i.p.) injection of scrapie resulted in scrapie infectivity that was first detected in the spleen [92,93,94]. Within the spleen and other LRS tissues, PrP^Sc^ has been shown to accumulate on follicular dendritic cells (FDCs) and within tingible body macrophages (TBM, [95,96]). Elimination of FDCs results in a failure of prions to establish infection in secondary LRS tissues and suggests that FDCs actively replicate PrP^Sc^ while TBMs scavenge PrP^Sc^ [96,97,98]. Accumulation of PrP^Sc^ on FDCs has been shown to occur for the duration of prion infection and does not result in cellular dysfunction [99,100,101,102]. Evidence suggests that PrP^Sc^ produced by FDCs can infect sympathetic nerves that innervate LRS tissue, as denervation or increasing the physical distance between FDCs and nerve fibers can extend the incubation period of disease or prevent disease transmission [103]. Following i.p. inoculation, prions were found to spread from the spleen to the thoracic spinal cord via the splenic sympathetic axons, with subsequent spread caudally and rostrally to the lumbar and cervical spinal cord, respectively, prior to detection in the brain (Figure 2A, [83,94,104]). In the brain, scrapie infectivity was first detected in caudal brain structures such as the medulla and the midbrain and eventually spread to the cerebellum and the cerebral cortex. 

Per os infection of prion agent results in neuroinvasion along defined anatomical pathways that involve the sympathetic and parasympathetic pathways innervating the gut. Intragastric prion inoculation of mice resulted in detection of infectivity in the Peyer’s patches of the gut, followed by spread of infectivity via the myenteric plexus and the enteric nervous system (ENS) to the thoracic spinal cord and finally the brain (Figure 2B, [105]). Consistent with this pathway of prion transport, splenectomy did not prolong the incubation period following intragastric inoculation. In sheep naturally infected with scrapie, PrP^Sc^ immunoreactivity was first detected in the Peyer’s patches. Later in the disease course, PrP^Sc^ immunoreactivity was detected in the myenteric plexus and ENS, the celiac sympathetic ganglia near the thoracic spinal cord, subsequently in the intermediolateral cell column of the thoracic spinal cord, and finally in the brain [106]. Simultaneously, PrP^Sc^ immunoreactivity was also found in the parasympathetic nodose ganglia and subsequently in the dorsal motor nucleus of the vagal nerve (DMNV). Oral BSE challenge of cattle also identified PrP^Sc^ immunoreactivity and infectivity that spread from the ENS to the brain via sympathetic pathways involving the thoracic spinal cord and parasympathetic pathways involving the vagus nerve [107,108,109]. Oral inoculation of hamsters with 263K scrapie prions recapitulated many of the findings from the sheep studies where 263K PrP^Sc^ was detected in the ENS, then in the sympathetic ganglia, thoracic spinal cord, and later in the medulla [110,111,112]. Importantly, scrapie was also observed to invade the CNS through the DMNV independent of the thoracic spinal cord pathway [113,114]. In this case, scrapie prions were hypothesized to invade the ENS, then travel through the vagal nerve parasympathetic pathway, directly invade the DMNV, and subsequently transneuronally spread throughout the CNS (Figure 2B). Neuroinvasion via the DMNV is also observed in deer per os inoculated with CWD [115,116]. Overall, these data indicate the retrograde spread of PrP^Sc^ through the autonomic nervous system via sympathetic and parasympathetic pathways after extraneural inoculation.

### 2.3. Intraneural Inoculation of Prions

Prions spread along defined neuroanatomical pathways following inoculation into peripheral nerves [117,118]. Unilateral inoculation of scrapie prions into the sciatic nerve of hamsters resulted in a significantly shortened incubation time compared to all other inoculation routes with the exception of intravenous and intracerebral routes [117]. Importantly, scrapie infectivity was detected in spinal cord segments at earlier timepoints post-infection compared to other extraneural infection (intraperitoneal, oral, subcutaneous, etc.) routes suggesting that scrapie prions were able to bypass the spleen and other LRS tissues, and directly invade and spread via axonal networks (Figure 2C). Consistent with these studies, a detailed analysis of the temporal and spatial spread of hyper (HY) PrP^Sc^ following inoculation of the sciatic nerve revealed that prions initially spread to ipsilateral ventral motor neurons in the lumbar spinal cord followed by transport along the rubrospinal, corticospinal, vestibulospinal, and reticulospinal descending motor pathways [118]. Axons of these four motor pathways synapse either directly or indirectly on VMNs [119]. HY PrP^Sc^ was detected in progressively more rostral spinal cord segments starting with the T10-T13 segment corresponding to where the sciatic nerve enters the lumbar spinal cord (Figure 2C). Within the brain, PrP^Sc^ was detected in the ventral portion of the contralateral RN (origin of the rubrospinal tract) which decussates and has direct projections to ventral motor neurons found in the lumbar spinal cord associated with the inoculated sciatic nerve. Other regions that are associated with the sciatic nerve motor pathways were also affected including the contralateral hindlimb motor cortex (origin of the corticospinal tract) and the ipsilateral lateral vestibular nucleus (origin of the vestibulospinal tract). Since HY PrP^Sc^ was not detected in the spleen following sciatic nerve inoculation, neuroinvasion and subsequent spread was via the sciatic nerve and not from sympathetic innervation from the spleen [118,120]. In addition to HY, sciatic nerve inoculation experiments with drowsy (DY) or 139H strain of prions, resulted in transport of PrP^Sc^ along the same four descending motor pathways [119,121]. Intralingual inoculation of HY has demonstrated PrP^Sc^ deposition in the hypoglossal nucleus, consistent with PrP^Sc^ spread via the hypoglossal nerve [122]. Overall, PrP^Sc^ is transneuronally transported along defined neuroanatomical pathways in the anterograde (intraocular inoculation) or retrograde (intraperitoneal, intragastric, or intraneural) direction. This is also independent of prion strain, suggesting this is a common feature of prions. A summary of the discussed transport pathways can be found in Table 1.

## 3. Cell Types Involved in Prion Transport

While it is well-established that prions invade and spread through the peripheral nerves to access the CNS, the mechanism by which this phenomenon occurs has remained elusive. Neurotropic viruses such as rabies and pseudorabies travel from the periphery to the CNS via axons. For example, the rabies virus utilizes microtubule polymerization as it undergoes retrograde transport along motor neuron axons [123]. Herpes viruses also utilize microtubules to spread along both sensory and motor neurons [124]. Since neurotropic viruses and prions spread along established neuroanatomical pathways after infection, the processes utilized by these viral agents serve as a starting point to elucidate the mechanisms involved in prion transport. 

**Figure 3:**
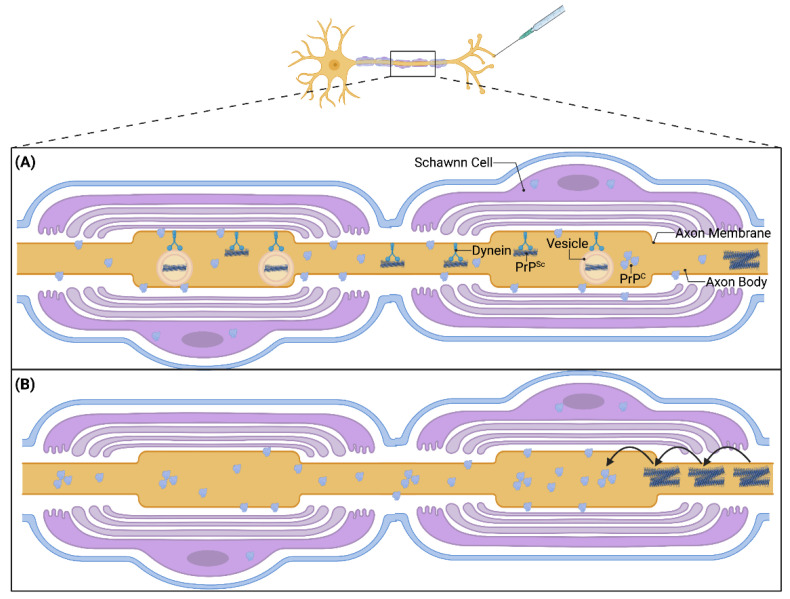
Prion Transport Mechanisms. Current theories of PrP^Sc^ axonal transport after neuroinvasion include retrograde PrP^Sc^ transport on dynein motors (**A**) in a traditional axonal transport paradigm. Of note, PrP^Sc^ may be free aggregates or contained within membranous vesicles such as endosomes or lysosomes. A “domino-like” mechanism has also been proposed (**B**), where PrP^Sc^ initiates the conversion of neighboring PrP^C^, and this conversion chain continues down the length of the axonal projection. Image created with BioRender.com, accessed on 28 February 2022.

The exact cellular location of PrP^Sc^ within neuronal pathways is unknown. In the nervous system, PrP^C^ is expressed not only in neurons and axons, but it has also been found in Schwann cells that myelinate axons in the PNS, oligodendrocytes that myelinate axons in the CNS, as well as astrocytes and microglia that support CNS neurons [16,125]. PrP^Sc^ was localized to the adaxonal membrane in peripheral nerves of scrapie-infected sheep [126] and was also described in adaxonal location in peripheral nerves of CJD patients after immunohistochemical analyses [127], implicating neuronal axons as a pathway of PrP^Sc^ spread. In addition, PrP^Sc^ aggregates traveled along neuritic projections in in vitro neuronal cultures [128]. To complement this finding, Schwann cells with ablated PrP^C^ expression using two different transgenetic mouse models did not slow the progression and incubation period of scrapie after intraperitoneal inoculation in mice [129,130]. This further strengthens the overarching finding that prions invade the CNS through peripheral neuronal pathways and utilize axons to propagate along these pathways towards central structures. These studies also suggest that the contribution of Schwann cells and oligodendrocytes are not required for PrP^Sc^ transport. There is still speculation on the exact location of PrP^Sc^ within an axonal segment. PrP^C^ has been noted as a GPI anchored membrane protein [13], but its conversion into PrP^Sc^ with altered properties may translocate it to another area on the axon, and confocal laser scanning imaging has detected PrP^Sc^ on the axonal periphery and within the axonal cytoplasm [131]. PrP^Sc^ populations have also been localized to endosomal and lysosomal compartments (Figure 3A), suggesting that PrP^Sc^ may also utilize intracellular membranous vesicles for movement within cells [128,132]. Interestingly, it was found that only a small fraction of PrP^Sc^ was associated with membrane-bound vesicles, indicating free PrP^Sc^ is also present within the cell. These findings suggest different roles of PrP^Sc^ based on axonal location and cell compartment where one population of PrP^Sc^ is primarily transported to other structures while another population aggregates and causes disease pathology. Alternatively, this may indicate multiple modalities of PrP^Sc^ transport that utilize different subcellular mechanisms.

## 4. Prion Transport Rate and Machinery

The velocity of PrP^Sc^ transport and whether prions utilize a fast or slow mode of axonal transport may provide insight into the mechanisms of prion transport. Axonal transport includes two molecular motors, kinesin and dynein. Kinesin is the main component involved in anterograde transport (from soma to synapse) while dynein takes part in retrograde transport (from synapse to soma). Axonal transport can also be divided into two main types, fast axonal transport and slow axonal transport which includes two components, a and b. Both transport mechanisms utilize kinesin and dynein motors and are differentiated based on their speeds, cargoes, subtypes of kinesin and dynein, and cofactors [133]. Fast axonal transport moves organelles and vesicles containing neurotransmitters at upwards of 400 mm/day. Slow axonal transport primarily moves cytoskeletal elements such as microtubules and neurofilaments (slow component a) or cytosolic proteins (slow component b) at less than 8 mm/day [134]. 

**Figure 4:**
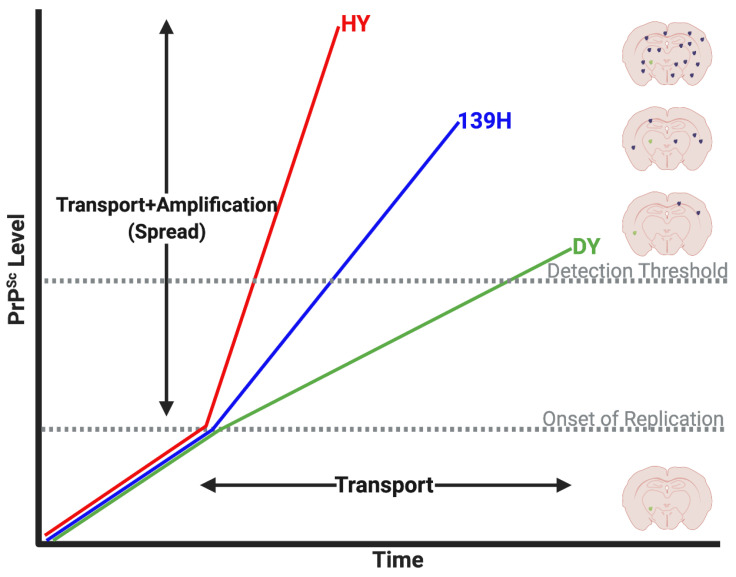
Prion Transport and Spread. PrP^Sc^ can traffic to its target directly (transport) or can convert endogenous PrP^C^ anywhere along the pathway and amplify (spread). Currently, detection methods such as immunohistochemistry (IHC) or Western blot (WB) can only measure the spread of PrP^Sc^ because threshold levels of PrP^Sc^ are required for successful detection. Moreover, this spread has been demonstrated to vary between prion strains and can influence calculation of transport/spread. More sensitive measurement techniques can approximate transport more accurately (requires lower PrP^Sc^ threshold for detection or even directly measure PrP^Sc^ transport). Note: Light green dots in coronal brain sections represents the original inoculum PrP^Sc^, while the black dots represent newly replicated PrP^Sc^. Image created with BioRender.com, accessed on 15 March 2022.

Estimates of the rate of prion spread is consistent with slow axonal transport mechanism (Figure 3, [104,117,118,119]). Importantly, prion spread was determined by assaying for prion infectivity via animal bioassay or immunodetection of PrP^Sc^. These assays do not discriminate between the original inoculum and newly converted prions, therefore, the time to detection of infectivity or PrP^Sc^ may be a combination of both transport and replication, hence the use of the term prion “spread” (Figure 4). Initial estimates of prion spread calculated from the detection of scrapie infectivity in different tissues at various times post infection determined that mouse adapted scrapie prions spread approximately 1–2 mm/day [104,117]. Subsequent studies corroborated that the spread of prions in the nervous system is consistent with slow axonal transport [85,112]. In a series of studies that utilized the sciatic nerve inoculation paradigm and three hamster-adapted rodent prion strains, strain-specific differences in the rate of PrP^Sc^ spread were identified. In these studies, the rate of spread of HY, 139H and DY PrP^Sc^ in the nervous system, as determined by immunohistochemistry, was 1.10 ± 0.11, 1.80 ± 0.27 and 4.14 ± 0.35 mm/day, respectively [119,121]. While these values were noted to be statistically different, they are still within the range of slow axonal transport. The reason for the observed differences is unknown, however these strains were shown to have differing rates of prion formation with the HY strain having a faster rate of prion formation compared to 139H and DY [34]. It is possible, that all three strains move along axons at the same velocity but since HY PrP^Sc^ amplifies faster than PrP^Sc^ from the other two strains, it may reach a level of abundance that is detectable by IHC faster than either DY or 139H (Figure 4). Alternatively, these findings could indicate that different strains utilize different host mechanisms of PrP^Sc^ transport. Overall, the actual velocity of PrP^Sc^ transport is unknown, as PrP^Sc^ may traffic to tissues much earlier in the disease process that can only be elucidated through experiments that directly detect inoculum PrP^Sc^. A summary of prion transport rate studies can be found in Table 1.

The role of endogenous PrP^C^ in the transport of PrP^Sc^ from the periphery to the CNS is poorly understood. Landmark studies confirmed the requirement of PrP^C^ for successful prion infection [135,136] since PrP^C^ expression is a necessity for prion formation. Studies utilizing PrP^C^ expressing neural grafts found that not only did the target CNS tissue require PrP^C^ for successful infection and pathology, but the peripheral pathways leading to the tissue also required PrP^C^ expression to propagate pathology from the periphery to the CNS [137,138]. This may indicate a need for a continuous supply of PrP^C^ from the site of inoculation to the target site in the CNS for successful infection and clinical pathology of CNS structures. It is possible that PrP^C^ is required for axonal transport or that PrP^C^ must be present along the whole tract to serve as a substrate for continual conversion of PrP^C^ to PrP^Sc^ all the way to the CNS in a “domino-like” fashion (Figure 3B). Alternatively, the failure to establish infection in the PrP^C^ containing grafts following peripheral inoculation may be due to the grafts not being synaptically connected to the peripheral neuronal pathways and therefore not allowing PrP^Sc^ to invade the graft. Interestingly, multiple cell culture studies have concluded that although endogenous PrP^C^ may play a role in initial PrP^Sc^ uptake into the cell, it is not necessary for infection of the cell or PrP^Sc^ transport between cells [139,140]. Evidence from imaging studies conducted on in vitro primary neuronal cultures found that PrP^C^ knockout neuronal cells could still take up and transport PrP^Sc^ within neurites [128] so PrP^Sc^ may not require endogenous PrP^c^ for transport.

## 5. Conclusions

Studies of the peripheral to central transport of prions have yielded many results of prion spread into the CNS. As evidenced by the studies above, prions spread along defined neuronal projections that innervate the inoculation site. Peripheral extraneural inoculation allows many prion strains to replicate in the LRS and travel along sympathetic projections toward the spinal cord where the agent spreads rostrally toward the brain. In the case of per os inoculation, prions spread along sympathetic and parasympathetic projections of the ENS and invade the spinal cord and DMNV, respectively. Peripheral intraneural injections demonstrate prion spread along defined neuroanatomical pathways that connect peripheral axonal pathways with specific brainstem nuclei and/or cortical areas. Currently, efforts are being undertaken to understand mechanisms behind prion spread. So far, PrP^C^ has been demonstrated to be a necessary part of successful prion infection, and evidence has indicated prions utilize axonal networks for peripheral to central transport. The approximate spread of prions is consistent with a slow axonal transport mechanism, yet the exact speed, subcellular mechanisms, and specific cofactors behind this transport phenomenon are still unknown. Overall, future research should focus on elucidating these mechanisms to further our understanding of peripheral prion disease pathogenesis.

## Figures and Tables

**Table 1:** Summary of prion pathogenesis experiments.

Inoculation Site	Studies	Route of Invasion	Neural Pathways	Targets	Transport	Rate
Intraocular	Buyukmihci et al., 1983 Fraser & Dickinson, 1985 Kimberlin & Walker, 1986 Scott & Fraser, 1989 Scott et al., 1992	Retinal Ganglion Cells	Optic Nerve Optic tract	SC LGN Visual Cortex	Anterograde	Slow 1.0 mm/day
Intraperitoneal	Kimberlin & Walker, 1979 Kimberlin & Walker, 1980 Kimberlin & Walker, 1982 Kimberlin & Walker, 1986 Kimberlin & Walker, 1989b	Spleen	ANS: Splenic Sympathetic Nerves	Brainstem	Retrograde	Slow 0.5–1.0 mm/day
Oral/Intragastric	Kimberlin & Walker, 1989a Beekes et al., 1996 Beekes et al., 1998 McBride & Beekes, 1999 Beekes & McBride, 2000 McBride et al., 2001 van Keulen et al., 2000 Sigurdson et al., 2000 Fox et al., 2006	Peyer’s Patches	ENS: Sympathetic and Parasympathetic (Vagus) Nerves	DMNV Brainstem	Retrograde	Slow 0.8–2.0 mm/day
Intraneural	Kimberlin et al., 1983 Bartz et al., 2002 Kratzel et al., 2007 Ayers et al., 2009 Langenfeld et al., 2016	Sciatic Nerve	Sciatic Nerve Lumbar Spinal Nerves	Lumbar VMNs RN Motor Cortex LVN RF	Retrograde	Slow 1.0–4.0 mm/day
Intralingual	Bartz et al., 2003	Hypoglossal Nerve	Hypoglossal Nerve	Hypoglossal Nucleus	Retrograde	ND

Abbreviations: ANS, autonomic nervous system; DMNV, dorsal motor nucleus of the vagus; ENS, enteric nervous system; LGN, lateral geniculate nucleus; LVN, lateral vestibular nucleus; ND, not determined; RF, reticular formation; RN, red nucleus; SC, superior colliculus; VMNs, ventral motor neurons.
